# Comment to “Ultrasonic Assessment of Females with Carpal Tunnel Syndrome Proved by Nerve Conduction Study”

**DOI:** 10.1155/2014/893963

**Published:** 2014-01-16

**Authors:** Daniele Coraci, Valter Santilli, Paola de Franco, Luca Padua

**Affiliations:** ^1^Board of Physical Medicine and Rehabilitation, Department of Orthopaedic Science, “Sapienza” University, Rome, Italy; ^2^Physical Medicine and Rehabilitation Unit, “Umberto I” polyclinic, Rome, Italy; ^3^Don Gnocchi Foundation, Piazzale Morandi 6, 20100 Milan, Italy; ^4^Institute of Neurology, Catholic University, 20121 Rome, Italy

We read with great interest the article written by Ajeena et al. “*Ultrasonic assessment of females with carpal tunnel syndrome proved by nerve conduction study*” [1].

The authors studied the correlation between the cross-sectional area (CSA) of median nerve at carpal tunnel inlet and the neurophysiological severity of the carpal tunnel syndrome (CTS). The authors recruited 35 females (63 hands) affected by different grades of CTS, diagnosed through neurophysiology; moreover, they enrolled, as control group, 40 women (80 hands) with no clinical/neurophysiologic evidences of CTS. The authors neurophysiologically divided the patients with CTS using Bland's scale [2], only considering three subgroups: mild (only sensory fibers involved, second grade of Bland's scale), moderate (sensory and motor fibers involved, third and fourth grade), severe (severe involvement of motor fibers, fifth and sixth grade). They studied median nerve with ultrasound from the distal forearm to the carpal tunnel outlet, measuring nerve CSA at the pisiform bone.

The results showed a mean CSA in CTS patients significantly greater than the CSA of control group and a different CSA between the three subgroups (worse the neurophysiological pattern, greater the CSA).

We agree with the authors about the consideration that ultrasound is a useful instrument for median nerve evaluation in CTS, but we have some comments on the study and its conclusion. On the basis of our experience, the measurement of the median nerve CSA in many points, from the forearm to the palm is very important. A correlation between CSA at wrist/palm and CSA at forearm is always suggested to discriminate a real enlargement of the nerve from a simple variation. Then, the compression of the median nerve at wrist is probably the most known condition in CTS, but an involvement may be located distally. The authors studied the nerve just to the outlet of carpal tunnel. The evaluation of median nerve dimension in the palm is useful to determine the exact site of the compression. This information is very important for surgical planning: many interventions do not resolve the syndrome because of a not exactly evaluated target.

Moreover, to our experience, the lonely CSA of median nerve is not enough. We consider the echotexture of the nerve very important. Median nerve in CTS usually presents a hypoechoic ultrasonographic pattern in the suffering sites. But in very severe and old CTS, the pattern is modified; that is, the nerve appears as more hyperechoic and sometimes with a CSA smaller than that of earlier CTS. This condition may be caused to a reshuffle of the nerve structures.

Finally, we consider ultrasound as a complementary and not a substitutive tool of the neurophysiology. To diagnose a CTS, a functional and morphological evaluation is advantageous. In this way, we can understand how much a large nerve is damaged. We found some example of big CSA with normal function of median nerve at wrist.

We can conclude that ultrasound reveals its powers after a good clinical and neurophysiologic evaluation.
